# Infectious events and prescription of antimicrobials in the coronary ICU

**DOI:** 10.1186/cc14171

**Published:** 2015-03-16

**Authors:** CE Bosso, SV Ferreira, GE Valerio, JV Moraes, V Raso

**Affiliations:** 1 Instituto do Coração de Presidente Prudente, Brazil; 2 Faculdade de Medicina - UNOESTE, Presidente Prudente, Brazil

## Introduction

The effectiveness of initially used antimicrobials represents an important factor in infectious events in coronary intensive care units (CICU) [1]. This study aimed to analyze the prevalence of infectious events and the prescribed antimicrobial in CICU.

## Methods

We analyzed the data of 2,005 patients admitted to the CICU for 3 years. The infectious events were based on general characteristics, main sites and outbreaks of infectious events in addition to the main microorganisms and pathogens. The prescription of antimicrobials was analyzed based on the isolated or associated use of antimicrobials. We also analyzed the adequacy of initial empirical antimicrobial according to the microbiological evidence. The general characteristics of events - that is, time, evidence of infection, infections by multidrug-resistant pathogens - are also presented.

## Results

The prevalence of infection was 4% (*n *= 81). Ventilator-associated pneumonia was 35% (*n *= 28), whereas urinary and primary bloodstream associated with catheters was 14% (*n *= 11) and 9% (*n *= 7), respectively. There was 82% (*n *= 66) evidence of microbiological infection. The main pathogens and microorganisms found were Gram-positive bacteria (*n *= 24, 30%; *Staphylococcus aureus *(*n *= 16, 20%), *Enterococcus faecalis *(*n *= 4, 5%), *Staphylococcus epidermidis *(*n *= 3, 4%)), Gram-negative (*n *= 43, 53%; klebsiella sp. (*n *= 13, 16%), *Pseudomonas aeruginosa *(*n *= 7, 9%), *Escherichia coli *(*n *= 7, 9%)) and fungi (*n *= 5, 6%; candida sp. (*n *= 2, 3%), *Candida albicans *(*n *= 1, 1%), *Candida dubliniensis *(*n *= 1, 1%)). The commonly prescribed antimicrobials were piperacillin/tazobactam (*n *= 32, 40%), vancomycin (*n *= 30, 37%), polymyxin B (*n *= 23, 28%), cefepime (*n *= 16, 20%), meropenem (*n *= 12, 15%), cefuroxime (*n *= 8, 10%), ciprofloxacin (*n *= 6, 7%), tigecycline (*n *= 6, 7%), ampicillin (*n *= 5, 6%), clindamycin (*n *= 4, 5%), chloramphenicol (*n *= 4, 5%), oxacillin (*n *= 4, 5%) and others (*n *= 32, 28%). There was 75% (*n *= 46) infection during hospitalization in the unit. Approximately 32% of infections were caused by multidrug-resistant pathogens, although there was efficiency of 81% in the proper use of initial antimicrobials.

## Conclusion

We conclude that infection is prevalent even in CICU, and that the microbiological profile is quite diverse, as well as the antibiotics. This allows us to better understand the profile of this kind of unit.
